# Tier-based formalism for safety assessment of custom-built radio-frequency transmit coils

**DOI:** 10.1002/nbm.4874

**Published:** 2022-12-13

**Authors:** Bart Romke Steensma, Alireza Sadeghi-Tarakameh, Ettore Flavio Meliadò, Cornelis A. T. van den Berg, Dennis W. J. Klomp, Peter R. Luijten, Gregory J. Metzger, Yigitcan Eryaman, Alexander J. E. Raaijmakers

**Affiliations:** 1 Division of Imaging and Oncology, University Medical Center Utrecht, Utrecht, The Netherlands; 2 Center for Magnetic Resonance Research, University of Minnesota, Minneapolis, Minnesota, USA; 3 Department of Biomedical Engineering, Eindhoven University of Technology, Eindhoven, The Netherlands

**Keywords:** EM simulations, RF coils, safety factor, SAR, validation

## Abstract

The purpose of this work is to propose a tier-based formalism for safety assessment of custom-built radio-frequency (RF) coils that balances validation effort with the effort put in determinating the safety factor. The formalism has three tier levels. Higher tiers require increased effort when validating electromagnetic simulation results but allow for less conservative safety factors. In addition, we propose a new method to calculate modeling uncertainty between simulations and measurements and a new method to propagate uncertainties in the simulation into a safety factor that minimizes the risk of underestimating the peak specific absorption rate (SAR). The new safety assessment procedure was completed for all tier levels for an eight-channel dipole array for prostate imaging at 7 T and an eight-channel dipole array for head imaging at 10.5 T, using data from two different research sites. For the 7 T body array, the validation procedure resulted in a modeling uncertainty of 77% between measured and simulated local SAR distributions. For a situation where RF shimming is performed on the prostate, average power limits of 2.4 and 4.5 W/channel were found for tiers 2 and 3, respectively. When the worst-case peak SAR among all phase settings was calculated, power limits of 1.4 and 2.7 W/channel were found for tiers 2 and 3, respectively. For the 10.5 T head array, a modeling uncertainty of 21% was found based on ${B_{1}}^{+}$ mapping. For the tier 2 validation, a power limit of 2.6 W/channel was calculated. The demonstrated tier system provides a strategy for evaluating modeling inaccuracy, allowing for the rapid translation of novel coil designs with conservative safety factors and the implementation of less conservative safety factors for frequently used coil arrays at the expense of increased validation effort.

## 1 ∣ INTRODUCTION

Radio-frequency (RF) transmit coils are used to excite nuclear spins with electromagnetic (EM) radiation. As a result of the interaction between the RF-transmit field and the tissue of the subject, power is deposited in tissue, which can lead to local and global heating effects. To ensure the safety of the subject, the International Electro-technical Commission (IEC) has specified limits for local and global specific absorption rates (SARs^1^), which are also adopted by the US Food and Drug Administration.^2^ While global SAR can be safely estimated from average RF input power and the subject's weight, local SAR needs to be assessed through EM simulations.

A challenging aspect of local SAR assessment is determining the uncertainty in SAR estimation that arises from differences between the simulation model and the experimental results obtained from the physical implementation of the RF coil (modeling uncertainty, also referred to as modeling error^3^). The calculation of the modeling uncertainty is usually done by comparing measurements on a phantom with simulations in a validation procedure that ultimately assesses uncertainties in the coil model. A wide variety of validation methods have been described in the literature.^4-6^ The most important validation methods are ${B_{1}}^{+}$-mapping,^7^ MR thermometry,^8^ temperature measurements using temperature probes,^9,10^ and E-field measurements.^11^ In addition, scattering parameters and lumped element values can be compared between measurements and simulations. Several groups have published workflows in which complete coil validation strategies are outlined.^3,10,12-15^ However, these workflows typically present a single, very extensive validation procedure. Such extensive validation procedures have a great advantage because they aim to create models that most accurately capture the transmit EM characteristics of the RF coil in order to minimize the difference between simulation and measurement. However, for RF coil development and even preliminary imaging studies, these extensive validation requirements often impede progress. In addition, the superior accuracy that is reached by such extensive validation efforts is not required in all circumstances. In fact, depending on the stage within the process of RF coil development, the tradeoff between SAR overestimation and validation efforts may be different. For example, when comparing the efficiency of multiple coil designs in volunteers, it would require tremendous effort to accurately validate the simulation models of all coils, while at a later stage this is desirable for only one coil design (the one that performs best) in order to enable scanning with power limits that are realistic and not overly conservative.

This need for flexibility in validation efforts has been recognized by a related field of research: RF implant safety. Manufacturers of active implantable medical devices (pacemakers, neurostimulators) together with MRI vendors have set up a guideline^16^ that outlines four degrees of modeling and validation effort, called tiers. Each incremental tier provides higher accuracy (less overestimation) at the expense of more extensive modeling and validation efforts. In this work, we present a similar formalism for the safety assessment of custom-built RF coils. We introduce a tier-based formalism, where the validation effort increases with increasing tier level. At higher tier levels the modeling uncertainty can be decreased and peak local SAR (pSAR) overestimation reduced. By design of the tier system, a large modeling uncertainty at lower tier levels will result in more conservative limits on power deposition, which, while still useful for evaluating a coil's performance during development, may compromise performance for general use.

In the literature, three sources of uncertainty in pSAR assessment are identified: intersubject variation,^17-20^ power monitoring uncertainty, and modeling uncertainty.^3^ The modeling uncertainty is determined based on the comparison between the simulation and the measurement. If deviations are large, the modeling uncertainty is considered large and vice versa. The system that we propose in this work is a formalism that defines which validation methods need to be included when determining the modeling error at a given tier and the consequences this has on the magnitude of the modeling uncertainty.

The tier level descriptions with corresponding validation requirements and consequences for modeling uncertainty and safety factors are presented in Table 1. Each tier level is explained in more detail in the Methods section. To exemplify the proposed methodology, we performed a coil validation for two arrays: an eight-channel fractionated dipole array for prostate imaging at 7 T^21^ and an eight-channel bumped fractionated dipole array for head imaging at 10.5 T.^10,22^ The two different coil arrays were developed at different research sites, field strengths and used in MRI systems from different vendors. As a result, there are some slight deviations in the simulation methods and validation data available for the two coil arrays. Nevertheless, we show that the tier system is easy to use in both cases, which demonstrates the feasibility of implementing this methodology with results from various sites.

Finally, the presented RF safety assessment procedure includes a statistical calculation method for the propagation of all the individual sources of uncertainty. The resulting safety factor incorporating these sources of uncertainty is compared with the safety factor resulting from a previously published uncertainty propagation approach.^3^

## 2 ∣ PROPOSED CONCEPTS

### 2.1 ∣ Uncertainties in pSAR estimation and the safety factor

When estimating pSAR based on EM simulations, there is always uncertainty with respect to the situation during an MRI experiment. This uncertainty arises from three uncorrelated sources, which were identified by Boulant et al.^3^

#### 2.1.1 ∣ Intersubject variations

With in vivo imaging, the subject in the scanner will differ from the human model in the EM simulations in nearly all cases. Tissue distributions, body dimensions, and coil position will all be subject dependent. As a result of this, local SAR distributions and pSAR values will inevitably be different between simulations and the actual in vivo MRI examinations.^17-20,23^ The uncertainty that is introduced by subject-dependent variations can be estimated through RF simulations on multiple human body models. The calculation of a distribution of possible pSAR values is possible by exploring multiple human models and RF shims.

#### 2.1.2 ∣ Power monitoring uncertainty

Power monitoring is an essential aspect of SAR estimation, as both local and global SAR calculations need an estimate of the time-averaged accepted power. Power monitoring uncertainties on a single transmit channel in a multitransmit configuration can lead to local differences between expected and actual SAR when these uncertainties translate into actual power monitoring errors (uncertainty describes the likelihood of errors). They can even cause a global scaling of SAR when power monitoring errors are present on all channels.

The method for monitoring power and determining its uncertainty can vary depending on the site and vendor. Some vendors might already provide estimates of uncertainty in the power monitoring, or use additional safety margins in their power monitoring system to account for the estimated uncertainty. In other cases, sites may have self-built power monitoring systems in place for which the uncertainty can be determined. The exact method of determining accepted power and and power monitoring uncertainty is not a main focus of this work and is left up to the user. At our site, average forward and accepted power can be monitored at the scanner for every transmit channel by using bidirectional couplers to sample forward and reflected power.^3,24^ The power measurements provided by these directional couplers are calibrated by an MR physicist from our site on a regular basis. The calibration is performed with a power meter and corrects for losses between the amplifier and the coil due to cables and connectors. For this specific procedure, uncertainties in power monitoring are introduced by the limited precision of the bidirectional couplers and the uncertainty of the peak power measurements.

#### 2.1.3 ∣ Modeling uncertainties

If the coil or the phantom model deviates from the actual characteristics of the implemented coil and phantom, simulated EM fields will be different from those measured in the scanner. Such modeling errors can result from many factors, including differences in coil geometry, coil loading, differences in coil materials, feed structures, matching networks, scattering parameters, or the effects of cables. Also, differences in the phantom properties or geometry could contribute to the modeling uncertainty. In total, these errors comprise the modeling uncertainty for a given coil. Modeling uncertainties can be quantified by comparing measurements with simulations on a phantom with known dielectric composition and geometry, a process typically referred to as simulation validation.^25^ In earlier work, the modeling uncertainty was found by calculating the root-mean-square error between measurements and simulations.^3^ Apparent modeling uncertainties could also arise from errors in the ${B_{1}}^{+}$ or temperature measurements themselves, thus contributing to the modeling uncertainty, although these errors do not arise from problems with the simulation model.

Note that often the terms “errors” and “uncertainties” are used interchangeably. In this work, we make a distinction where we call “error” an actual deviation, for example, a deviation between simulated and actual quantities. On the other hand, we consider “uncertainty” as the likelihood of making errors.

### 2.2 ∣ Uncertainty propagation and calculation of the safety factor

To avoid that uncertainties in the EM simulation or power monitoring result in 10-g-averaged pSAR underestimation, the estimated pSAR can be multiplied by a safety factor that takes into account all the previously described uncertainties. We first define the 10-g-averaged pSAR that is estimated during the MR examination, based on EM simulations and power monitoring on the scanner. Throughout this manuscript, this pSAR value will be referred to as ${\text{pSAR}}_{\text{est}}$. To arrive at a corrected pSAR value $pSAR_{est,corr}$ that results in an acceptably low probability of underestimating the pSAR during the MRI examination, the following calculation is carried out:

(1) $${\text{pSAR}}_{\text{est,corr}}={\text{pSAR}}_{\text{est}}*\text{safety factor}.$$

The above mentioned safety factor should minimize the risk of underestimating pSAR during an MR examination, given the fact that there are uncertainties in the simulation and power monitoring. The safety factor is therefore defined as

(2) $$Safety\;factor=1+\frac{\Delta pSAR_{est}}{pSAR_{est}}.$$

In Equation 2, the relative uncertainty of the simulated pSAR assessment is represented by $\frac{\Delta pSAR_{est}}{pSAR_{est}}$. This “overall” uncertainty can be calculated by separately identifying the different sources of uncertainty (power monitoring uncertainty, intersubject variation modeling, and modeling uncertainty) and propagating them into an overall uncertainty.

${\text{pSAR}}_{\text{est}}$ is a product of the normalized simulated pSAR (${\text{pSAR}}_{\text{sim}}$ normalized to 1 W total input power) and the total average input power $P_{\text{in}}$ that can be measured on the scanner.


(3) $${\text{pSAR}}_{\text{est}}=P_{\text{in}}*{\text{pSAR}}_{\text{sim}}$$

The relative uncertainties in power monitoring and ${\text{pSAR}}_{\text{sim}}$ can be reasonably assumed as uncorrelated and therefore they can be propagated using the standard root-sum-of-squares rule for error propagation^26^:

(4) $${\left(\frac{\Delta pSAR_{est}}{pSAR_{est}}\right)}^{2}={\left(\frac{\Delta P_{in}}{P_{in}}\right)}^{2}+{\left(\frac{\Delta pSAR_{sim}}{pSAR_{sim}}\right)}^{2}$$

Uncertainty in the pSAR simulation can be a result of intersubject variation and modeling uncertainty; these uncertainty terms are again assumed to be uncorrelated and will propagate as^26^:

(5) $$\Delta pSAR_{sim}^{2}=\Delta pSAR_{sim.int.subj.var}^{2}+\Delta pSAR_{sim.model.uncert.}^{2}$$

Substituting Equation 5 into Equation 4 yields

(6a) $${\left(\frac{\Delta pSAR_{est}}{pSAR_{est}}\right)}^{2}={\left(\frac{\Delta P_{in}}{P_{in}}\right)}^{2}+\frac{\Delta pSAR_{sim.int.subj.var}^{2}+\Delta pSAR_{sim.model.uncert.}^{2}}{pSAR_{sim}^{2}}$$

(6b) $${\left(\frac{\Delta pSAR_{est}}{pSAR_{est}}\right)}^{2}={\left(\frac{\Delta P_{in}}{P_{in}}\right)}^{2}+{\left(\frac{\Delta pSAR_{sim.int.subj.var.}}{pSAR_{sim}}\right)}^{2}+{\left(\frac{\Delta pSAR_{sim.model.uncert.}^{2}}{pSAR_{sim}}\right)}^{2}$$

Once the total uncertainty is found by propagating the different uncertainties, the final safety factor can be calculated with Equation 2.

### 2.3 ∣ Tier system

If there are large uncertainties in the pSAR estimation, the safety factor should be large, which results in a conservative estimation of corrected pSAR. Uncertainties in pSAR estimation can be reduced by a thorough validation of EM simulations, which can reduce the modeling uncertainty. However, depending on the intended use of an RF coil, it might not always be feasible or desirable to perform a full validation of EM simulations. To provide some flexibility in how to determine modeling uncertainties, we propose a tier-based validation formalism to stratify the amount of effort required for RF coil validation.

In the proposed tier system, the validation effort increases with increasing tier level. At higher tier levels the modeling uncertainty can be decreased and pSAR overestimation reduced. By design of the tier system, a large modeling uncertainty at lower tier levels will result in more conservative pSAR calculations. The tier system stratifies only the amount of effort put into determining the modeling uncertainty. Although we provide examples on how to include uncertainty caused by intersubject variation, RF shims, and power monitoring uncertainty, this is explicitly not part of the tier system. The following tier levels are defined:

Tier 1: no RF simulations are performed. All RF power is assumed to be dissipated in 10 g of tissue leading to a very limited power budget. This tier level is the same as the approach presented by Vignaud et al.^27^ Because this tier level is already extremely conservative, we do not make use of a safety factor that corrects for power monitoring uncertainty.

Tier 2: RF simulations are performed and ${B_{1}}^{+}$-maps are used for validation. In this case, the modeling uncertainty may be calculated by comparing the magnitude of ${B_{1}}^{+}$-measurements and simulations on a voxel-by-voxel basis. The calculation of the safety factor, which is multiplied with the peak SAR found from the simulation, is based only on validation with ${B_{1}}^{+}$-maps. However, because no validation of the metric to be controlled (temperature or pSAR) is performed, an additional buffer is added to the safety factor. Ideally, we would have an estimate of how large pSAR deviations will be in the case of specific ${B_{1}}^{+}$ uncertainties. The assessment of such relations is likely coil-specific and is omitted in this manuscript. Our approach is to multiply the safety factor from a tier 2 safety assessment by a factor of 2. As demonstrated later in this manuscript, this approach leads to a safety factor that exceeds the safety factor as calculated with a tier 3 validation. We would like to emphasize that determining this additional buffer for tier 2 is left up to individual sites and regulatory bodies. In case no SAR validation is performed, the final safety factor is determined as

(7) $${\text{safety factor}}_{\text{final}}=\text{buffer}*\text{safety factor}.$$

Tier 3: RF simulations are performed and a validation procedure is performed by MR thermometry measurements, as well as ${B_{1}}^{+}$-mapping, which mainly serves as an additional consistency check. The modeling uncertainty is calculated using a voxel-by-voxel comparison of measured and simulated field distributions. The ${B_{1}}^{+}$-map and temperature map will result in different modeling accuracies, where the largest of the two is used for further calculations.

Specifically for parallel transmit systems using RF coil arrays, the modeling error in tiers 2 and 3 can be different depending on the RF shim that is used in the validation measurements. To alleviate this problem as much as possible, validation data can be acquired with multiple different RF shim settings.

The tier system does not specify any guidelines for how well scattering parameters should match, but it is known from the literature that simulations will match better to experimental results when the scattering parameters match.^28^ Potential differences in scattering parameters between simulations and measurements will result in larger deviations between simulated and measured ${B_{1}}^{+}$ field distributions, which, as outlined in on 2.2, will result in a larger modeling error and thus larger safety margins. Because of this, we explicitly do not provide any additional penalty for a mismatch in scattering parameters.

Table 1 shows the three tier levels, the steps that are required to achieve these tier levels, and the consequences for the modeling uncertainty.

For tier 1, no quantitative validation effort is required, which results in very conservative limits on average input power. For tier level 2, the validity of the model is checked by comparing magnetic fields obtained through simulations and experimentally. However, because the validity of the SAR and the temperature simulations is not verified, the safety factor is still going to be relatively conservative, so for our example we multiplied the safety factor by an additional factor of 2. Only in tier 3, when both a ${B_{1}}^{+}$-map and spatial temperature maps are measured, can the modeling uncertainty be determined completely from the validation effort.

### 2.4 ∣ Determining the modeling uncertainty

As part of the tier-based safety assessment formalism, we propose a new method to determine the modeling uncertainty based on spatial maps of SAR and ${B_{1}}^{+}$ from the simulation and measurement. A voxel-wise difference is calculated for the two maps in the following way:

(8) $$\hat{{\text{SAR}}_{\text{diff}}}(x)=\frac{{\text{SAR}}_{\text{meas}}(x)-{\text{SAR}}_{\text{sim}}(x)}{{\text{SAR}}_{\text{sim,}\max}}$$

(9) $${\hat{B_{1}^{{+}^{2}}}}_{\text{diff}}(x)=\frac{|{B_{1}^{+}}_{\text{meas}}(x){|}^{2}-|{B_{1}^{+}}_{\text{sim}}(x){|}^{2}}{|{B_{1}^{+}}_{\text{sim,}\max}(x){|}^{2}}$$

In Equations 8 and 9, ${\text{SAR}}_{\text{sim}\max}$ and ${{B_{1}}^{+}}_{\text{sim},\max}$represent the maximum SAR and ${B_{1}}^{+}$ values in the simulated volume or slice. As peak SAR determines the final safe limits of operations, peak values are used as a reference. For both the head and the body array, dipole antennas were used that reach the highest SAR values in the central slice where the current on the conductor is highest. In the examples provided in this work, only the central slice was used to determine the modeling uncertainty. In the case of different array configurations, where pSAR values can be located outside of the central slice and the location is not clearly defined, a comparison of volumes instead of slices is recommended. The distribution of the voxel-wise difference can be plotted in a histogram, in which the positive error is determined as that which is not exceeded in 99.9% of all voxels. This value is set as the modeling uncertainty. Note that the maximum positive error over all voxels could be used as the modeling uncertainty instead. However, the exact value of the maximum positive error in the distribution will probably lead to a statistically unrealistic value because the tail of the underlying statistical distribution is not well sampled and there might be outliers in the measurement due to, for example, image artifacts at the edges of the phantom. Therefore, we take the positive error that is not exceeded in 99.9% of all voxels. This is in line with the work of Meliadò et al.^18^ on the pSAR_99_ value: the modeled pSAR value that is not exceeded in 99.9% of all subject and RF shim combinations. An important factor in determining the modeling uncertainty is the resolution of the validation measurements and simulations. In this work, we resampled the simulations at the (lower) resolution of the measurements. Specifically for the estimation of pSAR from MR thermometry maps, we ensured that the resolution of the acquired data did not exceed 1 cm^3^, so that the simulated pSAR (averaged over 10 g) could be compared with the pSAR derived from the MR thermometry measurement, which is expected to be spatially smooth because of heat conduction during the experiment.

## 3 ∣ METHODS

To exemplify the tier system methodology, we calculated power limits for all tier levels for an eight-channel transmit array of fractionated dipole antennas, to be used for prostate imaging at 7 T. The safety factor and power limits were calculated for a use case where RF phase-only shimming is performed on the prostate and for a use case with random RF phases. Additionally, a tier 2 validation was performed for an eight-channel bumped dipole array for head imaging at 10.5 T.^10,22^ For both arrays, we did not calculate power limits for scenarios in which phase-magnitude shimming is applied, as the numbers for intersubject variation were not available for the two coil arrays included in the manuscript. To calculate the safety factors, the modeling uncertainty, intersubject variation, and power monitoring uncertainty were determined based on data available from work on previous manuscripts.^10,22^

### 3.1 ∣ Modeling uncertainty

#### 3.1.1 ∣ Body array

Measurements were performed on a 7-T MRI system (Philips Achieva, Philips Healthcare, Best, The Netherlands). Validation data were acquired on a phantom filled with polyvinylpyrrolidone^29,30^ (1000 g/l) and salt (2.6 g/l), resulting in electrical and thermal properties ε_r_ = 37, σ = 0.4 S/m, and C_p_ = 4000 J/(kg*K). ${B_{1}}^{+}$-maps were acquired with the actual flip angle method (3D, resolution 1.52 x 1.52 x 10.0 mm^3^, field of view 438 x 438 x 30 mm^3^, and TE/TR1/TR2/FA: 2.2 ms/50 ms/250 ms/65°).^31^ Four different RF shims were used, one RF shim with all transmit phases set to 0 (0 phase mode), one RF shim with neighboring transmit phases alternating between 0 and 180 degrees (0/180 phase mode), and two RF shims with a ${B_{1}}^{+}$ hotspot in, respectively, the left (left mode) and right (right mode) side of the phantom.

For the same RF shims, MR thermometry was performed using the proton resonance frequency shift (PRFS) method.^8^ For the MR thermometry experiments, an average power of 20 W/channel at the coil was used. To achieve RF heating, a 4.5-ms, 9.1-μT block pulse was given as a prepulse in advance of each imaging excitation pulse (duty cycle 10%, peak power of heating pulse 200 W, total heating time 6 min) with an off-resonance of 100 kHz to avoid interference with the imaging sequence.^32^ A 2D multislice acquisition with a resolution of 2.88 x 2.88 x 6.00 mm^3^, a field of view of 414 x 414 x 42 mm^3^, and a TE/TR/FA: 10 ms/15 ms/11° were used to acquire the PRFS thermometry maps. During the MRI acquisition, per channel forward and reflected power were monitored using bidirectional couplers (EME 7020/30A, EME-HFTechnik). After the temperature map was calculated from the MR thermometry measurements, a spatial smoothing was performed in 2D using a 3 x 3 Gaussian kernel to remove spurious artifacts. To estimate SAR from the temperature maps, a linear fit ($SAR=C_{p}\frac{\Delta T}{\Delta t}$) was determined for the first minute of the heating series where temperature was increasing linearly.

The antenna array and the phantom were modeled in Sim4Life (Zurich Med Tech, Zurich, Switzerland). Measurements were compared with simulations to obtain the modeling uncertainty. To obtain a good match between transmit phases on the scanner and in the simulation, the simulations were corrected by adding a global phase shift to the individual channels to compensate for phase offsets between simulated and measured channels. This was carried out by comparing and matching relative transmit phases of the individual channels from a series of eight gradient echo images acquired with the individual transmit antennas. To do so, the transmit phase of the individual channels was varied until the structural similarity (as calculated in Matlab 2018b; MathWorks, Natick, MA, USA) between the simulated and measured transmit phase map was minimal. The results of this procedure are shown in Figure S1.

After coregistration of the measured and simulated ${B_{1}}^{+}$ and SAR maps, the modeling uncertainty was determined using the method described in the Proposed Concepts section. Because a total of eight values were available from the four different RF shims, the ${B_{1}}^{+}$-maps, and the SAR maps, in every comparison the worst case value was used as the final modeling uncertainty. The final dimensions of the coregistered ${B_{1}}^{+}$ and SAR maps were 50*80 voxels, resulting in 4000 datapoints being included in the modeling uncertainty analysis.

#### 3.1.2 ∣ Head array

Validation measurements for the head array were performed on a 10.5-T MRI system (Siemens magnetom; Siemens Healthineers, Erlangen, Germany).^10^ Data were acquired in a cylindrical jar-shaped phantom filled with hydroxyethyl cellulose gel (14 g/l) and NaCl (2.9 g/l), resulting in electrical properties ε_r_ = 78 and σ = 0.5 S/m. ${B_{1}}^{+}$ maps were acquired with the actual flip angle method^31^ (3D acquisition, resolution 2 x 2 x 5 mm^3^, field of view 256 x 208 x 260 mm^3^, and TE/TR1/TR2/FA: 3 ms/15 ms/75 ms/60°) using four different transmit phase settings: a circularly polarized (CP) shim, a linear mode, a random mode, and a zero-phase mode. Experimental assessment of increases in temperature was performed using temperature probes. Because it was not possible to make a spatial comparison with simulations over a complete slice with probe measurements, the temperature probe results are not included in this work.

Phantom measurements were compared with EM simulations, which were performed in HFSS (Ansys, Canonsburg, PA, USA). To obtain a good match between the simulations and the measurements, and consistency of the input phases on the scanner and in the simulation, the measured and simulated scattering matrices were matched using circuit cosimulations.^10,28^ More details on the validation measurements for the head array can be found in the work of Sadeghi-Tarakameh et al.^10^

Once again, the modeling uncertainty was calculated by determining the spatial difference between the simulated and measured ${B_{1}}^{+}$-maps and finding the difference which was only exceeded in the top 0.1% of voxels. The worst-case modeling uncertainty of the four RF shims was used as the final modeling error. The final dimensions of the coregistered ${B_{1}}^{+}$ maps were 97*97 voxels, resulting in 9409 datapoints being included in the modeling uncertainty analysis.

### 3.2 ∣ EM simulation setup and determining intersubject variation

#### 3.2.1 ∣ Body array

Simulation results from a study by Meliadò et al.^18^ were used to find intersubject variation for 7-T prostate imaging in human models. For clarity, the methods described in^18^ are briefly outlined in this section. A database of 23 human models was generated from Dixon scans acquired at 1.5 T with a mockup antenna array positioned around the pelvis to achieve realistic deformation of the body. EM simulations of the eight-channel fractionated dipole array were performed on all these body models. An approximate grid resolution of 2 mm was used in all models (minimum discretization step 0.7 mm, maximum step 4 mm). After exporting 10-g-averaged Q-matrices from all models, virtual observation points (VOPs)^33^ were extracted for rapid SAR calculations over many RF shims.

pSAR values were calculated for RF shims with varying input phases over all channels and input power divided equally over all channels. In one case, RF shims were chosen randomly, and in another case RF shims were chosen to achieve a high ${B_{1}}^{+}$ in the prostate. In the latter case, variation in the input phases was achieved by calculating the optimum RF phase for all volunteers testing out each of these different optimum phases on all the volunteers individually.^18^ This procedure resulted in two histograms of peak SAR values that included intersubject variation and also variation in RF shim phases. A gamma-distribution was fitted to the histogram by using commercially available software (histfit function, Matlab 2019b; MathWorks). Based on the gamma distribution, a pSAR value can be estimated that is expected not to be exceeded in 99.9% of all examinations (the pSAR_99_ value). A pSAR_99_ value was calculated for the two histograms: one with completely random shim phases and one with shim phases that achieve a high ${B_{1}}^{+}$ in the prostate. From the histograms, we can also calculate the average pSAR value over all shim sets, $\bar{pSAR}$. After calculating the pSAR_99_ and the $\bar{pSAR}$, uncertainty in local SAR estimation because of intersubject variation is calculated as

(10) $$\frac{\Delta {\text{pSAR}}_{\text{int.subj.var.}}}{\text{pSAR}}=\left(\frac{{\text{pSAR}}_{99}}{\bar{\text{pSAR}}}-1\right)*100\%.$$

The intersubject variation was calculated for the two scenarios where either RF phase shimming on the prostate was performed, or a scenario with completely random RF phase shims. A scenario where input power is also varied over the different channels is not considered in this manuscript. In this specific example, not only intersubject variation is included, but also the effect of different RF shims. Although these are in principle two separate sources of uncertainty, they are both included in the intersubject variation in this example. This was done mainly because from the available data as presented in the paper, it is not possible to separate between the two sources of uncertainty.

#### 3.2.2 ∣ Head array

Simulation results from Sadeghi-Tarakameh et al. were used to find simulated SAR values for the 10.5-T head array.^10^ EM simulations were performed in HFSS on two human models from the virtual family^10,34^ and two models of the CST voxel family. For the head models, an approximate grid resolution of 2 mm was used (minimum 2 mm, maximum 2.08 mm). pSAR was calculated for the CP mode in all models. Intersubject variation was calculated by calculating the lowest and the highest pSAR in the models. Because of the smaller size of the database and the number of included RF shims, intersubject variation was calculated in a slightly different way for the head array than for the body array.

### 3.3 ∣ Power monitoring uncertainty

For the 7-T body array, forward and reflected power were monitored on a per channel basis using bidirectional couplers (EME 7020/30A, EME-HFTechnik). For the 10.5-T head array, forward and reflected power were measured with 50-dB directional couplers (Werlatone Inc., New York, NY, USA) using LMR-400–type cables. To obtain the same per channel input power in the simulations and the measurements, for the body array the input powers measured in the scanner with the bidirectional couplers were provided as an input to the simulation. For the head array simulations, cosimulations were used to match the simulated and measured S-parameters,^28^ after which the same forward powers were provided in the simulations as in the measurements. For the 7-T array, the calibration inaccuracy of the bidirectional couplers in combination with the uncertainty of the power meter that was used during calibration of the bidirectional couplers was set as the power monitoring uncertainty. For the 10.5-T brain array, a vendor-provided number was used for the power monitoring uncertainty.

### 3.4 ∣ Calculation of safety factors, ${\text{pSAR}}_{\text{est},\mathrm{corr}}$, and power limits

After the pSAR uncertainties were determined, Equations 6b and 2 were used to calculate the safety factor. For the body array, the safety factor was calculated for two scenarios: one with random RF phase shimming and one with RF phase shims that achieve a high ${B_{1}}^{+}$ in the prostate. For the head array, the safety factor was calculated for the CP mode. For RF shims with input power varying over different channels—which are not included in the current study—the pSAR uncertainty would have to be calculated again.

From the simulations described in Meliadò et al.^18^ and Sadeghi-Tarakameh et al.,^10^ the average pSAR over all models was calculated. This was performed for the body array with RF phase shimming and random RF phase shims, as well as for the CP mode in the head array. Based on the ${\text{pSAR}}_{\text{est}}$ value and the pSAR uncertainty, the safety factor and ${\text{pSAR}}_{\text{est,corr}}$, can be calculated. From these values, it is possible to determine average power limits for the described shimming scenarios. Because power is divided equally over all channels, both total and per channel power limits are provided.

## 4 ∣ RESULTS

### 4.1 ∣ Modeling uncertainty

#### 4.1.1 ∣ Body array

**FIGURE 1:**
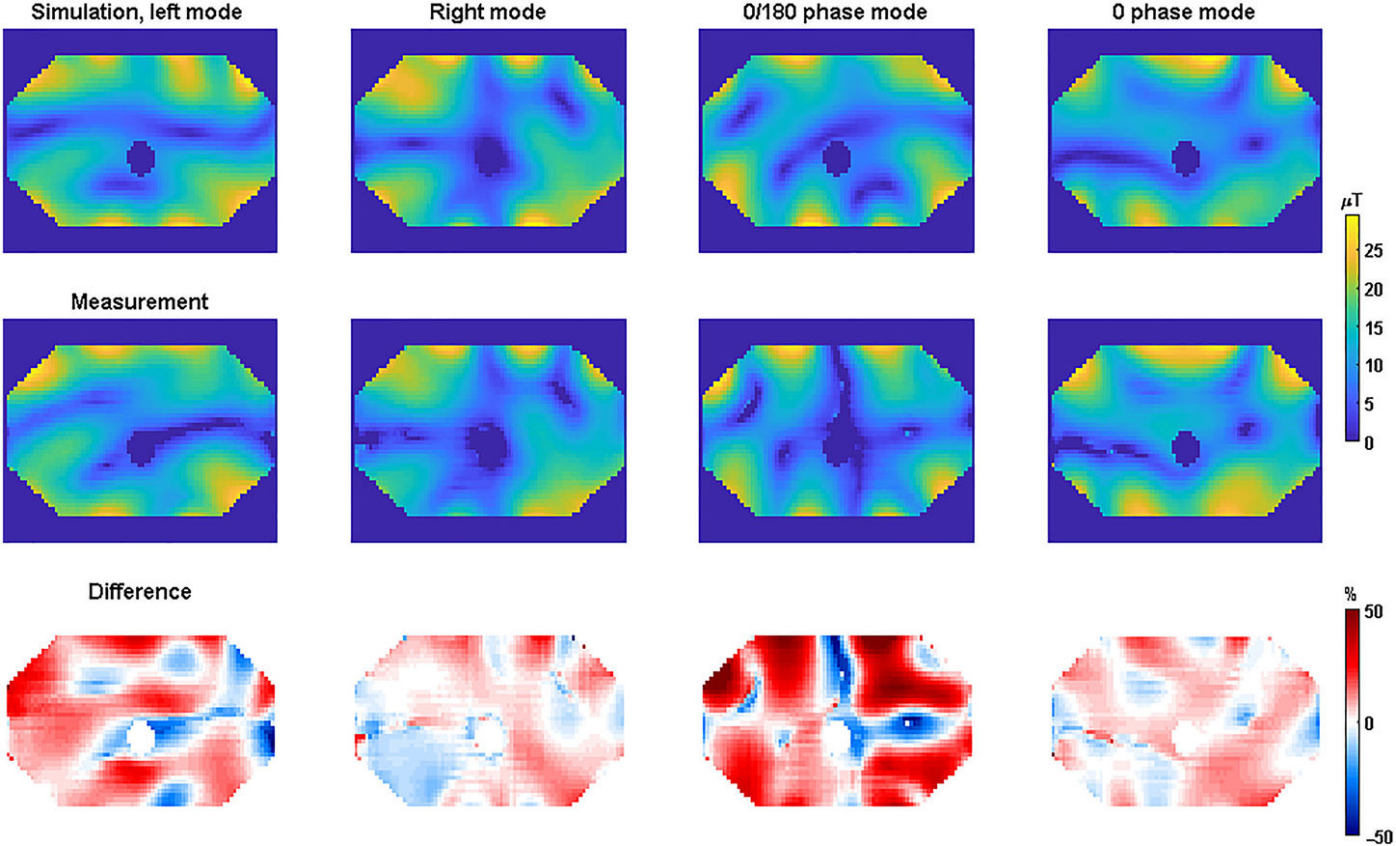
Simulated (top) and measured (middle) ${B_{1}}^{+}$-magnitude maps for the 7-T body array. The relative difference between the two was calculated per voxel as (B_1,meas_ − B_1,sim_)/B_1,sim,max_. Difference maps calculated using Equation 9 are shown in the bottom row

To determine the modeling uncertainty for the body array, measurements were compared with simulations for four different RF shims. For the different RF shims (left mode, right mode, 0/180 phase mode, and 0 phase mode), ${B_{1}}^{+}$-maps and MR thermometry maps were acquired. ${B_{1}}^{+}$-maps were acquired with the AFI method.^31^ The measured and simulated ${B_{1}}^{+}$-maps are shown in Figure 1. The difference maps were calculated according to Equations 8 and 9.

**FIGURE 2:**
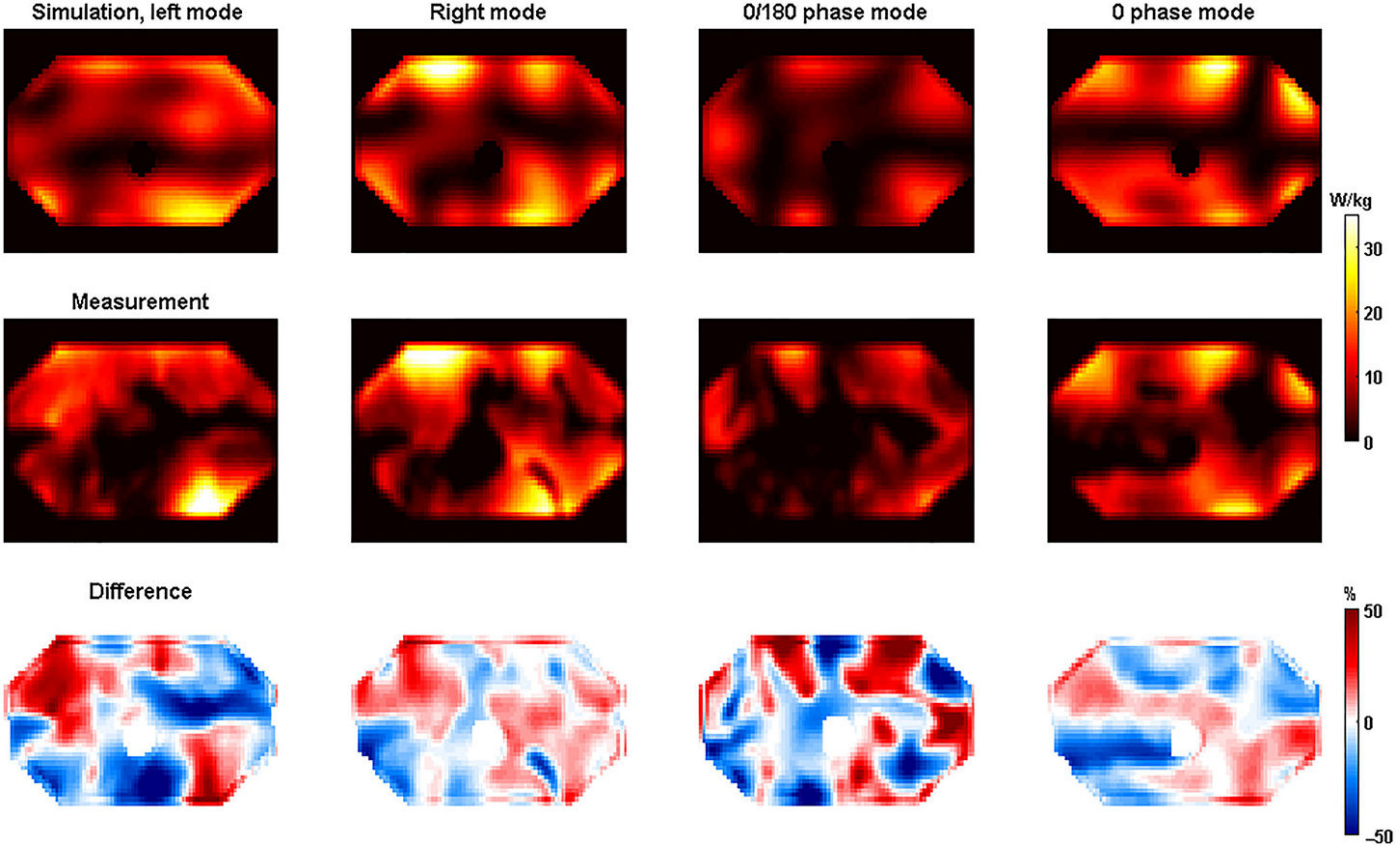
Simulated (top) and measured (middle) SAR-maps for the 7-T body array. The relative difference between the two was calculated per voxel as (SAR_,meas_ − SAR_,sim_)/SAR_,sim,max_. Difference maps calculated using Equation 8 are shown in the bottom row. SAR, specific absorption rate

The simulated and measured ${B_{1}}^{+}$-maps show excellent agreement for the right and 0 phase mode. For the “left mode” and the “0/180 phase mode”, quantitative deviations are somewhat larger. Figure 2 shows the same results, but for SAR as derived from the simulations and temperature measurements.

**FIGURE 3:**
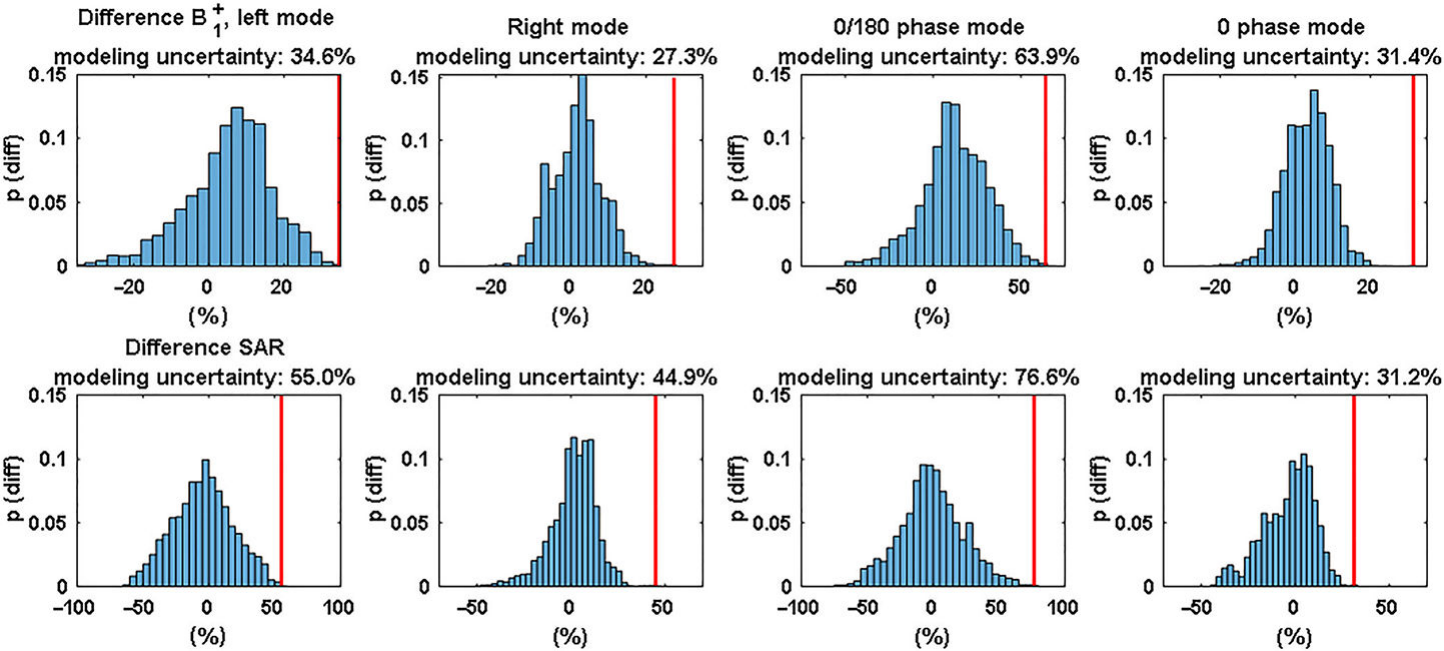
Histograms of the relative ${B_{1}}^{+}$ (top) and SAR (bottom) differences for the 7-T body array, using the data of the spatial difference maps shown in Figures 1 and 2. The red bars indicate the modeling uncertainty. SAR, specific absorption rate

Agreement between simulations and measurements was also demonstrated for the SAR maps. Similar to the ${B_{1}}^{+}$ measurements, the largest deviations were again found for the left mode and the 0/180 phase mode. The voxel-wise spatial differences are plotted in histograms for both the ${B_{1}}^{+}$ and the SAR measurements in Figure 3.

By finding the relative difference that exceeded the top 0.1% of all voxels, the modeling uncertainty was calculated. The modeling uncertainty is indicated by the red line in the plots. In general, larger modeling errors were found for the SAR comparison than for the ${B_{1}}^{+}$ comparison. The largest modeling error was found for the 0/180 phase mode (63.9% from ${B_{1}}^{+}$, 76.6% from SAR). There is no clear relationship between the height of the modeling error as calculated from ${B_{1}}^{+}$ compared with the modeling error calculated from the SAR.

#### 4.1.2 ∣ Head array

**FIGURE 4:**
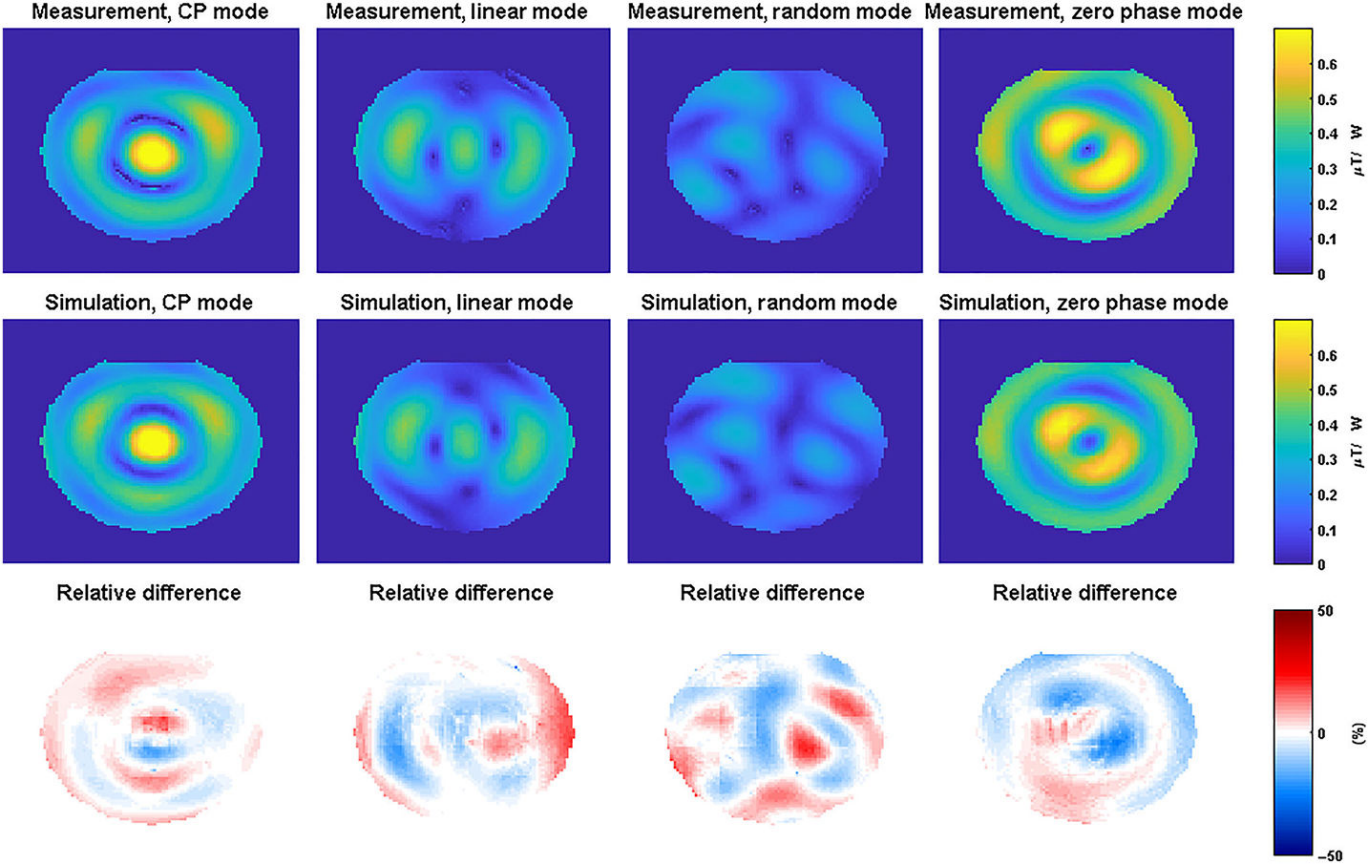
Measured (top row) and simulated (middle row) ${B_{1}}^{+}$-magnitude maps for the 10.5-T head arrays in a cylindrical gel phantom. The bottom row shows the relative difference between measurements and simulations calculated using Equation 9, CP, circularly polarized

To determine the modeling error for the head array, a tier 2 validation was done by comparing only ${B_{1}}^{+}$-maps. ${B_{1}}^{+}$-maps were acquired with four different RF excitation vectors (using the actual flip angle method^31^) and compared with simulations with the same input phases. The results of the validation procedure are shown in Figure 4.

**FIGURE 5:**
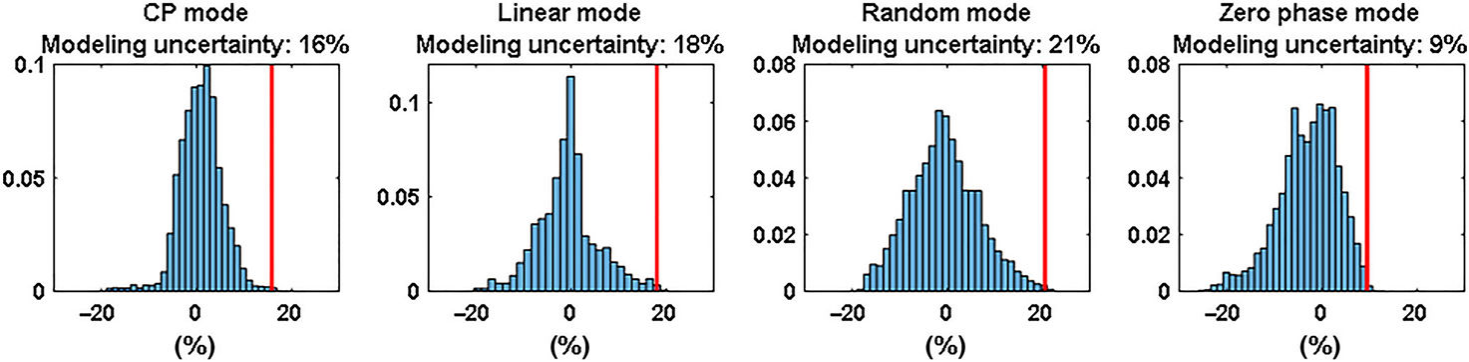
Histograms of the modeling errors for the 10.5-T head array, for all the different RF excitation schemes. CP, circularly polarized; RF, radio-frequency

Good qualitative agreement between the measurements and simulations can be observed in Figure 5 for all RF shims. Please note that in the original measurements and simulations there are rather large errors at the edges of the phantom, which are caused by voxels in the measurement that appear to have a high ${B_{1}}^{+}$ but are located just outside of the phantom. To remove these errors, the two first voxels on the edge of the phantom were removed through image erosion. This had no effect on the errors that were observed in voxels located inside the phantom. The distributions of the modeling errors are plotted in histograms in Figure 5.

Modeling errors are comparable between the different simulations, ranging from 9% (0 phase mode) to 21% (random mode). The worst-case modeling error, 21% for the random mode, is used as the modeling error to calculate the safety factor.

### 4.2 ∣ Intersubject variations

#### 4.2.1 ∣ Body array

**FIGURE 6:**
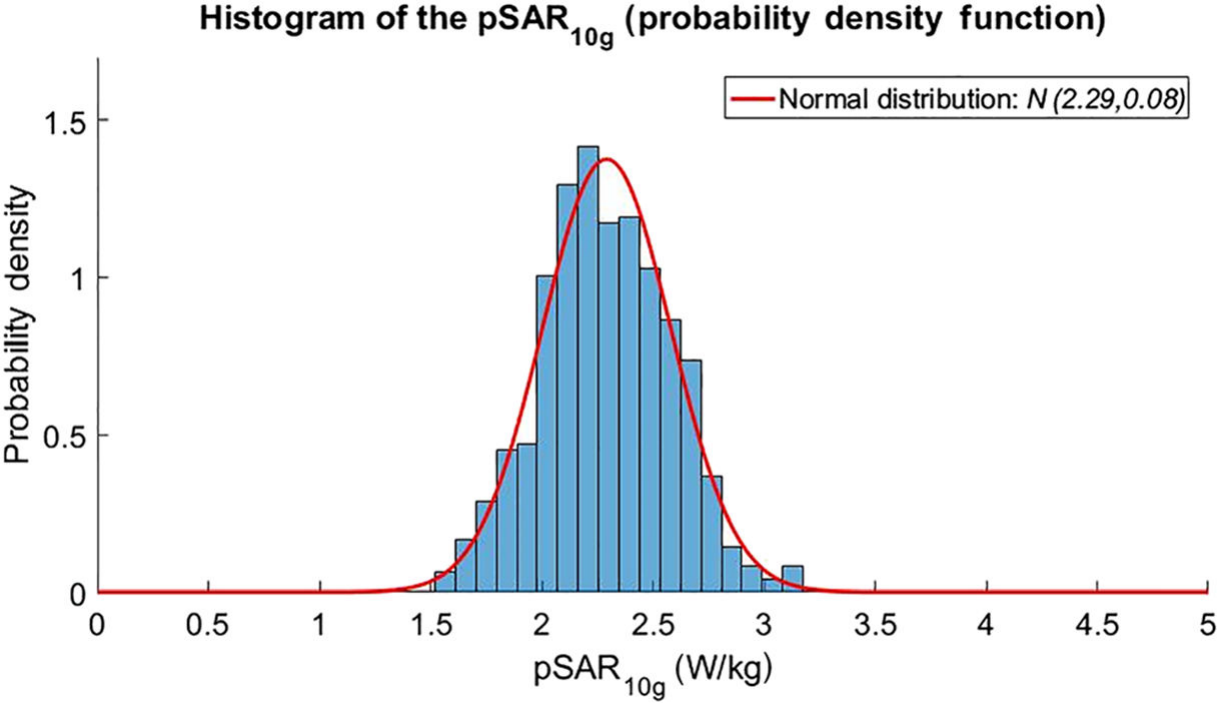
Histogram of 10-g averaged pSAR values for different subjects and shim values for the 7-T body array.^18^ The average pSAR value is 2.3 W/kg, while the pSAR value that is not exceeded for 99.9% of all cases is 3.2 W/kg. pSAR, peak local specific absorption rate

Based on the work of Meliadò et al.,^18^ an average pSAR of 2.3 W-kg was found for an RF shim that is optimized for prostate imaging. This pSAR value is averaged over the whole database of 23 models. To achieve this pSAR value, a total input power of 8 W was used, divided equally over all eight channels. The results of this analysis are shown in Figure 6.

To estimate intersubject variation in this case, we compare the average pSAR over all subjects (2.3 W/kg) with the simulated pSAR value that is not exceeded for 99.9% of all RF shims (the pSAR_99_ value, not to be confused with the modeling uncertainty); this pSAR_99_ value is 3.2 W/kg. This difference results in a relative intersubject variation of $\frac{\Delta pSAR_{int.subj.var.}}{pSAR}=39\%$. In the case of random RF shims, an average pSAR of 3.6 W/kg is found and a relative intersubject variation of 77%.

#### 4.2.2 ∣ Head array

In the work of Sadeghi-Tarakameh et al.,^10^ SAR simulations were performed on four human models in CP mode. For an input power of 8 W, pSAR values of 2.6, 3.0, 2.4, and 2.8 W/kg were found in simulations. In this database, the average pSAR over all models is 2.7 W/kg. By comparing the highest pSAR value (3.0 W/kg) and the lowest pSAR value (2.4 W/kg), an intersubject variation of 27% was found. Please note that this approach is not completely consistent with the approach for the 7-T body array, but because the database of human models was much smaller for the 10.5-T head array, this more conservative method was chosen to find the intersubject variation. Because simulations were performed for a single RF shim (CP mode), we only calculate the safety factor for the CP mode.

### 4.3 ∣ Power monitoring uncertainty

For the 7-T system (body array), the bidirectional couplers have a reported calibration accuracy of ~0.25 dB at 300 MHz, resulting in an uncertainty of 6%. For the 7-T system, an uncertainty of 10% was included to account for the instrumentation uncertainty of the power monitoring unit that was used to calibrate the bidirectional couplers (Giga-tronics 8542C Universal Power Meter). By adding both uncertainties in a sum-of-squares manner, a total uncertainty of 12% was obtained. For 10.5 T, the overall tolerances of the RF supervision system provided by the vendor were 12%.

### 4.4 ∣ Calculation of safety factors and power limits for different tier levels

#### 4.4.1 ∣ Body array

For the tier 1 approach it is assumed that all the power is deposited in 10 g of tissue. For an eight-channel array with 1-W input power per channel, this leads to a SAR of 800 W/kg. Taking into consideration a pSAR limit of 20 W/kg for body imaging in the first level controlled mode, the power limit will be 0.025 W/channel or 0.2 W total power.

For the validation in tiers 2 and 3, simulation and validation results are required. For tier 2, a modeling error of 64% was found by comparing the measured ${B_{1}}^{+}$-map with the simulations. An intersubject variation of 39% from the literature^18^ was used and the power monitoring error was 12%, as calculated from the specified accuracy of the directional couplers and the uncertainty of the calibration power meter. All these uncertainties add up to a total safety factor of 1.8, according to Equations 6b and 2. In addition, a scaling factor of 2 was used because validation by temperature was not performed. For tier 2, the simulated SAR was scaled from 2.3 W/kg to 2.3*1.8*2 = 8.3 W/kg. This value corresponds to an average input power of 8 W, or 1 W per channel. The power limit in the first level controlled mode (20 W/kg SAR limit) was therefore 20/8.3*8 ≈ 19.3 W total average power, or 2.4 W/channel. For tier 3, a modeling error of 77% was found when the same intersubject variation (39%) and power monitoring errors (12%) were used. A total safety factor of 1.90 was found, resulting in a corrected pSAR value of 2.3*1.9 = 4.4 W/kg for 8 W total input power. This results in a power limit of 36.4 W in total, or 4.5 W/channel. For the different tier levels, Table 2 lists which uncertainties are incorporated into the model and the resulting safety factors.

The intersubject variation of 39% that was found by Meliadò et al. is specifically calculated for a situation where RF shimming is applied, which results in very similar transmit phase distributions for every volunteer. In a situation where worst-case pSAR is considered for all possible phase shim settings, the same authors found a higher intersubject variation (77%). In this case, the average simulated pSAR is also higher (3.6 W/kg). This would be relevant if there is no demonstrated consistency between the RF shims provided on the scanner and in the simulation. In this case, the same calculations can be repeated with different numbers for simulated pSAR and intersubject variation. These calculations were performed and are shown in Table S1. The higher average pSAR values and higher intersubject variation result in total average power limits of 11.2 W (1.4 W/channel) and 21.6 W (2.7 W/channel) for tiers 2 and 3.

#### 4.4.2 ∣ Head array

If a tier 1 validation effort is performed, it is assumed that all the power is deposited in 10 g of tissue. For an eight-channel array with 1 W input power per channel, this leads to a pSAR of 800 W/kg. Considering the pSAR limit of 20 W/kg for head imaging in the first level controlled mode, the power limit will be 0.025 W/channel, or 0.2 W total power.

For the tier 2 validation, a modeling uncertainty of 21%, an intersubject variation of 27%, and a power monitoring uncertainty of 12% were calculated. These uncertainties are all incorporated in the total uncertainty budget, resulting in a final safety factor of 1.4. Also, because it was only possible to determine the modeling error from the ${B_{1}}^{+}$-maps, an additional scaling factor of 2 was used on top of the safety factor. The average simulated pSAR was 2.7 W/kg; however, after application of the safety factor and the losses in the transmit chain, the corrected pSAR was 7.4 W/kg. This resulted in a power limit of 2.6 W/channel, or 21.1 W total power. The modeling uncertainties and safety factors are provided in Table 3.

## 5 ∣ DISCUSSION

We have introduced a new tier-based formalism for safety assessment of custom-built RF coils or coil arrays. The tier system is inspired by the tier-based safety assessment that is used for active implants for MRI.^16^ Our proposed tier system provides flexibility between a more rigorous validation effort or a more conservative safety factor by stratifying the validation effort of pSAR assessment. Lower tier levels allow for more uncertainty in the pSAR estimation that will be propagated into a higher safety factor. The lower tier levels (1 and 2) can be used to scan volunteers earlier in the coil prototyping stages, where a large validation effort is not desirable, and safe scanning will be guaranteed by larger safety factors. Even although the power limits will be conservative, this enables researchers to compare multiple coil designs on volunteers while only having to perform EM simulations and using ${B_{1}}^{+}$ maps to determine safe power limits (if consistent with local institutional review board regulations).

The current implementation of tier 1 assumes that all power is deposited in 10 g of tissue, which results in extremely conservative power limits. Another possible implementation of tier 1 would be to calculate the worst-case pSAR for a given anatomy based on the volume integral equation method, for example, as in Guérin et al.^35^ This method would require the calculation of a full electromagnetic basis in the anatomy of interest, which is available in, for example, the head^36^ or the pelvis.^37^ The comparison of this implementation with the current tier 1 implementation is a subject for further investigation.

Within the framework provided by the tier system, it is not possible to perform a validation based on only the MR thermometry measurements. Because SAR is the metric of interest when determining the modeling error, one might argue that using only the MR thermometry-based validation would suffice for tier 3. However, as MR thermometry is generally more prone to errors and biases than ${B_{1}}^{+}$ mapping, obtaining ${B_{1}}^{+}$-maps is still very important as an additional consistency check and to provide more confidence in the MR thermometry data. For this reason, and because it is generally easier to acquire ${B_{1}}^{+}$-maps than MR thermometry maps, we did not include the option to validate based only on MR thermometry.

A scenario that is challenging to cover with the currently proposed tier system arises when a subject-specific body model is available. In this case, it would be reasonable to reduce intersubject variation to 0. The modeling error could be determined by ${B_{1}}^{+}$-mapping, but with current methods it would not be feasible to perform in vivo validation of SAR or temperature. A possible solution for this was recently proposed by Sadeghi-Tarakameh et al.,^38^ who proposed a Monte Carlo approach to determine the SAR modeling error from only ${B_{1}}^{+}$ validation. With further development of this method, a full validation based on comparison of only ${B_{1}}^{+}$-maps could be possible for subject-specific body models.

Recently, the International Society for Magnetic Resonance in Medicine (ISMRM) working group Best Practices for Safety Testing of Experimental RF Hardware published a consensus-based document^25^ that provides a complete description of safety testing for experimental RF hardware, including sections on EM simulations, validations of EM simulations, and uncertainty in pSAR estimation. The ISMRM document provides methods for determining the modeling uncertainty (or error) and for propagation of different uncertainties that can be utilized in our tiered approach.^3^ However, a flexible method for determining the modeling uncertainty and stratifying validation effort such as the tier system described in this work is not provided in the consensus document. The method that we use to determine the modeling uncertainty (by picking the error not exceeded by 99.9% of the voxels) follows the same principles as Meliadò et al.^18^ By providing a formal but also flexible method for RF coil validation, we hope to further contribute to a more standardized way to perform safety assessment of custom-built RF coils to accelerate coil development and human translation.

Our method provides flexibility in either assuming a conservative modeling uncertainty or striving for a more accurate determination of the modeling uncertainty by validating simulations with measurements. In the specific case of a 7-T fractionated dipole array for prostate imaging, the tier 2 and tier 3 validation resulted in average power limits of 2.4 and 4.5 W/channel when RF shimming was performed on the prostate. When random RF shims were used, higher average pSAR values and higher intersubject variation resulted in average power limits of 1.4 and 2.7 W/channel for tiers 2 and 3, respectively. For the body array, the power limits are the least stringent when a full validation with MR thermometry is performed. For the 10.5-T bumped fractionated dipole head array, a power limit of 2.6 W/channel was found for tier 2.

For the use cases that are described in this manuscript, pSAR was predetermined for specific RF shims (CP mode or focused on the prostate), or from the pSAR over many different RF shims. Another use case is when SAR is determined on the scanner using methods such as VOPs.^33^ In this case, it would still be possible to scale the simulated pSAR as calculated from the VOPs by a predetermined safety factor. Specific care should be taken in determining intersubject variation for RF shims with varying phase and amplitude, which is not possible with the simulation data that were used in this work. A problem with this use case is that the modeling error may vary over different RF shims, especially with the inclusion of RF amplitudes that vary per channel. This problem could be alleviated by introducing additional safety margins, which are already factored into VOPs (the overestimation factor). However, a thorough solution to this problem requires further investigation.

The tier system method was applied to two coil arrays based on available results from earlier work at two different research sites. Because the methods used by these sites were slightly different, the validation procedures are not completely consistent. For example, for the head array, only a tier 2 validation could be completed because no spatial temperature maps were available. Additionally, the method to calculate simulated pSAR and intersubject variation was different for the two coil arrays because there was a difference in the available simulation data. Finally, cosimulations were used in the simulations for the head array, but not for the body array. It has been demonstrated before that using cosimulations to match simulated and measured scattering parameters can improve the validation^28^ and thus can result in a reduction of the modeling error. By performing the tier method on these two different datasets, we hope to demonstrate consensus between research sites, as well as some flexibility on how to interpret and use the tier system.

Uncertainty in the assessment of pSAR is normally mitigated by applying a safety factor, which effectively increases the estimated pSAR, thus leading to more conservative average power constraints at the scanner. In earlier work,^9^ the overall safety factor was obtained by multiplication of the individual safety factors coming from different sources of uncertainty (intersubject variation, power monitoring uncertainty, and modeling uncertainty). That approach did not take into account the stochastic nature of these uncertainties and is overly conservative. We introduce an alternative way to calculate the safety factor from the different sources of uncertainty that is statistically justified. Note that the conventional uncertainty propagation method would result in a safety factor of 1.77 x 1.39 x 1.12 = 2.8, while the proposed approach results in a safety factor of 1.9 that translates into a reduction of the local SAR overestimation by 44%. To be able to propagate uncertainties using the sum-of-squares approach it is necessary that the different uncertainties are statistically uncorrelated. Because the underlying reasons for the different uncertainties (modeling uncertainties, intersubject variations, and power monitoring uncertainty) do not depend on each other, this assumption is reasonably justified.

Finally, we propose a new method to determine the modeling uncertainty based on a voxel-wise analysis of the differences between simulations and measurements. The modeling uncertainty is defined as the positive error that is not exceeded in 99.9% of all voxels. We take this specific margin because it is in line with earlier work that uses the same margin to assess pSAR^18^ and because it results in a conservative but not unnecessarily high safety factor. As demonstrated in the results, this leads to a relatively large and conservative modeling uncertainty, even when the measurements visually correspond very well to the simulations. Because of the way we calculate the modeling uncertainty, spatial maps are required for both temperature (or electric field) and ${B_{1}}^{+}$. In the specific case where thermal probe measurements are preferred over MR thermometry (e.g., when B_0_ drift is a problem) only a tier 2 validation procedure could be completed, such as is the case for the 10.5-T head array.

## 6 ∣ CONCLUSIONS

A formalism is presented for safety assessment of custom-built RF transmit coils that stratifies the level of simulation and validation effort in a tier system. The largest tier level provides minimum overestimation at the expense of considerable modeling and validation efforts, while it is also possible to put less effort into the modeling and validation, which results in more conservative limits on average power. The modeling uncertainty is determined by a statistical approach based on differences between simulated and measured ${B_{1}}^{+}$-maps/temperature maps. To prevent underestimation of pSAR, predicted pSAR levels are multiplied by a safety factor that is obtained by adding individual sources of uncertainty in the statistically appropriate sum-of-squares way. For tier 1 validation, a power limit of 0.025 and 0.0125 W/channel was found for the body array and the head array. For the 7 T body array, per channel power limits of 2.4 and 4.5 W/channel were found for tiers 2 and 3, when RF shimming was performed on the prostate. When using random RF shims, power limits of 1.4 and 2.7 W/channel were found. For the head array, the tier 2 validation resulted in a power limit of 2.6 W/channel when using the CP mode.

## Supplementary Material

Supplementary Figures

## Figures and Tables

**TABLE 1:** Different tier levels, the required simulation and validation effort, and the consequences for the modeling uncertainty and the resulting safety factor

Tier level	Required simulation and validation effort	Modeling uncertainty	Intersubject variation
Tier 1	No requirement	-	-
Tier 2	Simulation and validation by ${B_{1}}^{+}$-map	Determined from ${B_{1}}^{+}$-mapping error	Determined from literature or simulations
Tier 3	Simulation and validation by ${B_{1}}^{+}$-map and spatial temperature mapping	Determined from measurement, worst-case of spatial temperature mapping, and by ${B_{1}}^{+}$-mapping error	Determined from literature or simulations
Tier level	Power monitoring uncertainty	Additional scaling factor	Safety factor
Tier 1	-	-	No safety factor, all power is assumed to be deposited in 10 g of tissue
Tier 2	Determined from literature or measurements	2	Determined by validation effort	
Tier 3	Determined from literature or measurements	-	Determined by validation effort

**TABLE 2:** Different tier levels and the resulting modeling uncertainties and safety factors following different RF shimming approaches. The simulated pSAR value of 2.3 W/kg comes from the simulations of Meliadò et al^18^

Tier level	Modeling uncertainty	Inter subject variation	Power monitoring uncertainty	Safety factor	Additional scaling factor	Simulated pSAR	Corrected pSAR	Power limit
Tier 1	-	-	-	Not applicable	-	-	For 8 W P_in,_ peak SAR: 800 W/kg.	0.025 W/channel
Tier 2	64%	39%	12%	1.8	2	2.3 W/kg	8.3 W/kg	2.4 W/channel
Tier 3	77%	39%	12%	1.9	-	2.3 W/kg	4.4 W/kg	4.5 W/channel

Abbreviations: pSAR, peak local specific absorption rate; RF, radio-frequency.

**TABLE 3:** Different tier levels and the resulting modeling uncertainties and safety factors for CP mode

Tier level	Modeling uncertainty	Inter subject variation	Power monitoring uncertainty	Safety factor	Additional scaling factor	Simulated pSAR	Corrected pSAR	Power limit
Tier 1	-	-	-	Not applicable	-	-	For 8 W P_in_, peak SAR: 800 W/kg.	0.025 W/channel
Tier 2	21%	27%	12%	1.4	2	2.7 W/kg	7.6 W/kg	2.6 W/channel

Abbreviations: CP, circularly polarized; pSAR, peak local specific absorption rate.

## Abbreviations used:

EM: electromagnetic
IEC: International Electro-technical Commission
PRFS: proton resonance frequency shift
pSAR: peak local SAR
RF: radio-frequency
SAR: specific absorption rate
VOPs: virtual observation points
