# Supplementary material for: Tier-based formalism for safety assessment of custom-built radio-frequency transmit coils
Source: NMR Biomed. Author manuscript; Available in PMC 2023 Aug 9. (PMC10411033; doi:10.1002/nbm.4874)
Supplement: Supplementary Figures [file NIHMS1908999-supplement-Supplementary_Figures.docx]

**Supplementary material for review**


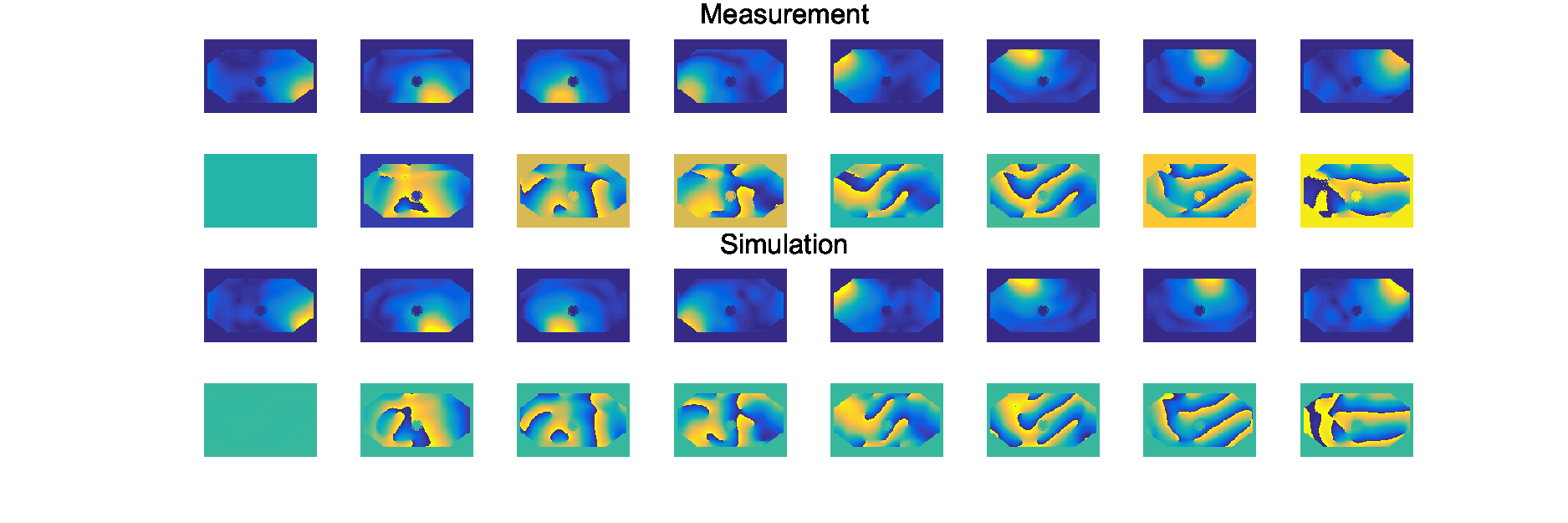


**Figure S1: single channel relative B_1_^+^-maps. The simulated B1+ phase distributions were corrected by adding a global phase shift to each individual channel to compensate for the phase delay between amplifier and coil array.**

| **Tier level** | **Modeling uncertainty** | **Inter subject variation** | **Power monitoring uncertainty** | **Additional scaling factor** | **Safety factor** | **Simulated pSAR** | **Corrected pSAR** | **Power limit** |
| --- | --- | --- | --- | --- | --- | --- | --- | --- |
| Tier 1 | - | - | - | - | No safety factor, all power is assumed to be deposited in 10g of tissue | - | For 8W P_in,_ peak SAR:  800 W/kg. | 0.025 W/channel |
| Tier 2 | 64% | 77% | 6% | 2 | 2.0 | 3.6 W/kg | 14.4 W/kg | 1.4 W/channel |
| Tier 3 | 77% | 77% | 6% | - | 2.1 | 3.6 W/kg | 7.5 W/kg | 2.7 W/channel |

**Table S2: different Tier levels and the resulting modeling uncertainties and safety factors following different approaches for random phase settings. Simulated pSAR value of 3.6 W/kg comes from simulations of Meliadò et al.**
